# Effect of extracorporeal CO_2 _removal on respiratory rate in spontaneously breathing patients with chronic obstructive pulmonary disease exacerbation

**DOI:** 10.1186/cc12066

**Published:** 2013-03-19

**Authors:** E Spinelli, S Crotti, L Zacchetti, N Bottino, V Berto, R Russo, M Chierichetti, A Protti, L Gattinoni

**Affiliations:** 1 Fondazione IRCCS Ca' Granda Ospedale Maggiore Policlinico, Milan, Italy

## Introduction

During severe exacerbation of chronic obstructive pulmonary disease (COPD) tachypnea, as a consequence of respiratory acidosis, and airflow limitation, due to small airway obstruction, lead to lung hyperinflation, respiratory distress and gas exchange impairment. Invasive mechanical ventilation could worsen lung hyperinflation and produce a vicious circle. We investigated whether increasing extracorporeal carbon dioxide removal (ECCO_2 _Cl) could reduce the respiratory rate (RR), so prolonging time for lung emptying and allowing resolution of hyperinflation.

## Methods

**Figure 1:**
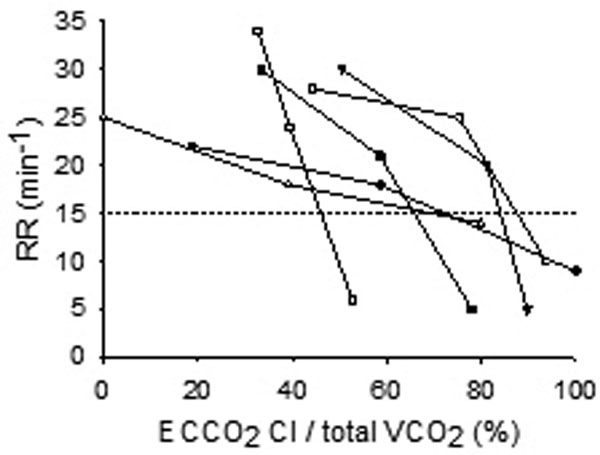


Six patients with COPD exacerbation with respiratory acidosis (PaCO_2 _83 ± 27 mmHg, pH 7.19 ± 0.1) and tachypnea (RR 39 ± 5) despite maximal non-invasive ventilation underwent venovenous extracorporeal membrane oxygenation (VV-ECMO). All patients were awake and spontaneously breathing an adequate air-oxygen mixture to correct hypoxemia (PaO_2 _72 ± 27 mmHg). While keeping the blood flow stable (2.9 ± 0.5 l/minute), we changed the gas flow of the artificial lung to modify the extracorporeal CO_2 _clearance as a percentage of total patient CO_2 _production (% ECCO_2 _Cl/total VCO_2_) and we observed the variations of RR. We recorded RR at three levels of gas flow in each patient (Figure 1).

## Results

In all patients RR decreased with the increase of extracorporeal CO_2 _removal and a negative correlation was found between RR and ECCO_2 _Cl/total VCO_2 _(*r*^2 ^= 0.42, *P <*0.01). In all patients we were able to obtain a reduction of RR below 15 (28 ± 4 vs. 8 ± 4, RR at low gas flow vs. RR at maximal gas flow, *P <*0.001). The selected maximal gas flow was variable between different patients (6.7 ± 2 l/minute), corresponding to different levels of ECCO_2 _Cl/total VCO_2 _(83 ± 17%, range 53 to 100%) and RR response (8 ± 4, range 5 to 14).

## Conclusion

In patients with COPD exacerbation, who failed noninvasive ventilation, VV-ECMO allows one to maintain spontaneous breathing. Titration of extracorporeal CO_2 _removal leads to control RR. This approach could interrupt the vicious circle of dynamic hyperinflation and allow the deflation of lung parenchyma.

